# Relationships between Intraocular Pressure, Effective Filtration Area, and Morphological Changes in the Trabecular Meshwork of Steroid-Induced Ocular Hypertensive Mouse Eyes

**DOI:** 10.3390/ijms23020854

**Published:** 2022-01-13

**Authors:** Ruiyi Ren, Anne A. Humphrey, David L. Swain, Haiyan Gong

**Affiliations:** Department of Ophthalmology, Boston University School of Medicine, Boston, MA 02118, USA; renruiyi@bu.edu (R.R.); humph2aa@cmich.edu (A.A.H.); dlswain@bu.edu (D.L.S.)

**Keywords:** steroid-induced ocular hypertensive mouse model, effective filtration area, trabecular meshwork, intraocular pressure, morphology, confocal microscopy, transmission electron microscopy

## Abstract

We investigated whether an inverse relationship exists between intraocular pressure (IOP) and effective filtration area (EFA) in the trabecular meshwork (TM) in a steroid-induced ocular hypertensive (SIOH) mouse model and the morphological changes associated with the reduction of EFA. C57BL/6 mice (*n* = 15 per group) received either 0.1% dexamethasone (DEX) or saline eye drops twice daily for five weeks. IOP was measured weekly. Fluorescent tracers were injected into the anterior chamber to label EFA at the endpoint. Injected eyes were fixed and processed for confocal microscopy. EFA in the TM was analyzed. Light and electron microscopy were performed in high- and low-tracer regions of six eyes per group. The mean IOP was ~4 mm Hg higher in DEX-treated than saline-treated control eyes (*p* < 0.001) at the endpoint. EFA was reduced in DEX-treated eyes compared to controls (*p* < 0.01) and negatively correlated with IOP (*R*^2^ = 0.38, *p* = 0.002). Reduced thickness of juxtacanalicular tissue (JCT) and increased abnormal extracellular matrix in the JCT were found to be associated with reduced EFA. Our data confirm the inverse relationship between EFA and IOP, suggesting that morphological changes in the JCT contribute to the reduction of EFA, thus elevating IOP in SIOH mouse eyes.

## 1. Introduction

Glucocorticoids are one of the most commonly prescribed classes of medications worldwide for the treatment of various ocular and systemic conditions, due to their broad-spectrum anti-inflammatory and immunosuppressive properties. However, as described for the first time in the 1950s [1], long-term usage of steroids can cause an elevation of intraocular pressure (IOP), the primary risk factor of glaucoma [2,3]. Steroid-induced glaucoma is a form of secondary open-angle glaucoma and clinically very similar to primary open-angle glaucoma (POAG). In both clinical conditions, the elevated IOP is due to an increased aqueous humor outflow resistance and is associated with morphologic and biochemical changes in the trabecular meshwork (TM) [4,5,6,7,8,9]. Steroids are known to induce ocular hypertension when administered via topical, periocular, and even systemic or inhalational routes. IOP rises after steroid therapy more frequently with topical administration than systemic administration [10]. Approximately 40% of the normal population have a measurable IOP increase after 4–6 weeks of topical steroid treatment and are considered to be “steroid responders”. In contrast, 95% of hypertensive POAG patients and 100% of low-tension POAG patients are “steroid responders” under treatment with topical steroid [11,12]. Non-glaucomatous steroid responders are at higher risk for developing POAG compared to non-glaucomatous non-responders [13,14], which suggests similar mechanisms in the pathology of steroid-induced ocular hypertension (SIOH) and POAG. Therefore, there is a need to better understand the mechanisms of SIOH.

To investigate the mechanisms of SIOH, various SIOH animal models have been developed in rabbits [15,16], cats [17], dogs [18], bovines [19], sheep [20], non-human primates [21], rats [22,23], and mice [24,25,26,27,28]. These models confirmed steroid-induced morphological and functional changes in the TM [23,24,27,29,30,31,32,33,34,35,36]. Previous SIOH mouse models also confirmed a reduction in outflow facility [24,25,28].

In the TM, experimental evidence suggests that the majority of outflow resistance is generated in the juxtacanalicular tissue (JCT) region [2,37,38]. A recent study also suggests that the increased flow resistance in glaucomatous eyes is generated immediately below the surface of the inner wall (IW) of Schlemm’s canal (SC) [39]. Therefore, we expect to find the most significant morphological differences in the TM of SIOH mouse eyes located in the JCT region, as shown by previous studies on SIOH mouse models [24,28].

Previous tracer studies concluded that aqueous humor outflow is “segmental” rather than uniform [40,41,42,43,44,45,46,47,48,49,50,51]. We termed the active fraction of the total area of the outflow pathway as effective filtration area (EFA) [46], which was previously analyzed by measuring percent effective filtration length (PEFL). Our group found a positive correlation between PEFL and outflow facility across three species (bovine, monkey, and human) with marked differences in the morphology of their outflow pathways [41,44,45]. Furthermore, a negative correlation was found between the TM PEFL and IOP in a secreted protein, acidic and rich in cysteine (SPARC)-null mouse model of ocular hypotension [52]. This study aims to determine whether an inverse relationship also exists between the PEFL and IOP in an ocular hypertensive model and to investigate what morphological changes contribute to the reduction of EFA. We utilized a recently reported mouse model of ocular hypertension induced by topical administration of dexamethasone (DEX) [27,53] to test our hypothesis that DEX will induce structural changes in the JCT region of the TM, resulting in a decrease in the EFA and, thereby, an increase in IOP.

## 2. Results

### 2.1. DEX Treatment Elevates IOP

**Figure 1:**
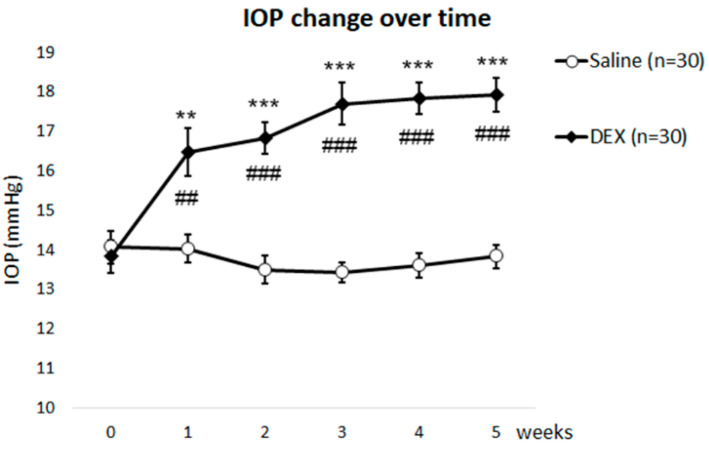
DEX Treatment Increased IOP. Dexamethasone (DEX) treatment increased Intraocular Pressure (IOP) over time. DEX-treated eyes showed significantly increased IOP at all time points after treatment compared to the saline-treated controls (**: *p* < 0.01; ***: *p* < 0.001). There was also a significant increase when compared to its own baseline (##: *p* < 0.01; ###: *p* < 0.001).

Unlike humans, all DEX-treated mice in this study appeared to respond to the steroid with ≥3 mm Hg increase in IOP in at least one eye. Because each eye responds differently to DEX treatment, each eye of a mouse can be measured as an independent data point for IOP [27]. The comparison of weekly IOP changes between two groups is summarized in Table 1. IOP significantly increased within a week and remained elevated for the following four weeks in the DEX-treated group compared to the saline-treated group (Figure 1), in which IOP remained unchanged for five weeks. IOP of DEX-treated eyes versus saline-treated eyes was 13.8 ± 0.4 vs. 14.1 ± 0.4 mm Hg at baseline (*p* = 0.69; two-tailed non-paired *t*-test), 16.5 ± 0.6 vs. 14.0 ± 0.3 (*p* = 0.001) after Week 1 of treatment, 16.9 ± 0.4 vs. 13.5 ± 0.4 (*p* < 0.0001) at Week 2, 17.7 ± 0.5 vs. 13.4 ± 0.3 (*p* < 0.0001) at Week 3, 17.8 ± 0.4 vs. 13.6 ± 0.3 (*p* < 0.0001) at Week 4, and 17.9 ± 0.4 vs. 13.8 ± 0.3 (*p* < 0.0001) at Week 5. When compared to its own baseline, the DEX group showed a significant increase after one week of treatment (*p* = 0.002 at Week 1, <0.0001 at Weeks 2–5; two-tailed paired *t*-test). In contrast, the saline group had no change in IOP over the five weeks of treatment when compared to its own baseline (*p* = 0.95 at Week 1, 0.33 at Week 2, 0.18 at Week 3, 0.37 at Week 4, and 0.61 at Week 5).

IOP change during five weeks of treatment was further analyzed by using three-way ANOVA with three factors: treatment (DEX vs. saline), time, and sex. The results showed that there was a significant main effect of the type of treatment (F (1, 56) = 83.88, *p* < 0.0001). There was also a significant main effect of time (F (5, 280) = 7.256, *p* < 0.0001) but no significant main effect of sex (F (1, 56) = 0.2556, *p* = 0.6151). Therefore, eyes from both sexes were combined for other analyses. There was also a significant interaction between the type of treatment and time (F (5, 280) = 11.18, *p* < 0.0001). No significant interaction was found between time and sex (F (5, 280) = 1.331, *p* = 0.2594) or between the type of treatment and sex (F (1, 56) = 1.163, *p* = 0.2855). There was no significant interaction between the type of treatment, time, and sex (F (5, 280) = 0.7144, *p* = 0.6131). These data suggested that DEX increased IOP significantly over time, while sex did not affect the IOP change.

### 2.2. DEX Treatment Reduces PEFL

**Figure 2:**
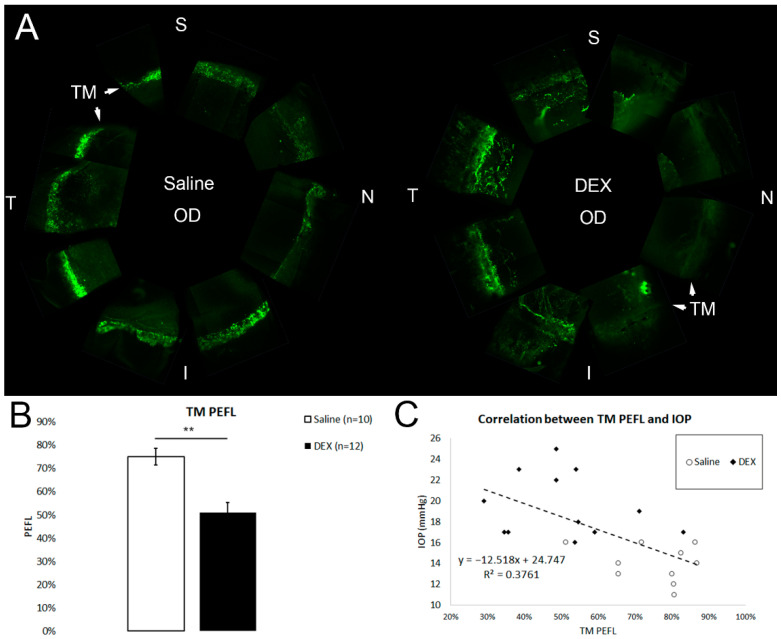
TM PEFL and its correlation with IOP. (**A**) Representative en face images of saline- and dexamethasone (DEX)-treated eyes (N: nasal; T: temporal; S: superior; I: inferior). The tracer distribution is shown in green. (**B**) DEX-treatment induced a significant reduction in trabecular meshwork percentage effective filtration length (TM PEFL) when compared to controls (**: *p* < 0.01). (**C**) There was a significant negative correlation between TM PEFL and IOP (*R*^2^ = 0.38, *p* = 0.002).

The en face images of the anterior segment showed the outflow pattern in each eye. Segmental flow was observed in both DEX-treated and saline-treated control eyes, but the flow pattern was more continuous in the control eyes when compared to DEX-treated eyes (Figure 2A).

When calculated by using en face confocal images, TM PEFL was 50.89 ± 4.52% in the DEX-treated group (*n* = 12), which was significantly less than the TM PEFL of 75.09 ± 3.60% (*n* = 10, *p* = 0.002; Mann-Whitney test) in the control group (Figure 2B). A significant negative correlation was found between TM PEFL and IOP (*n* = 22, *R*^2^ = 0.38, *p* = 0.002; linear regression) (Figure 2C).

**Figure 3:**
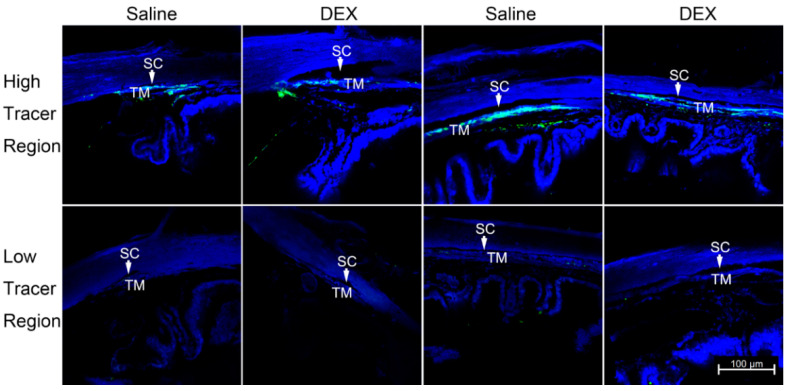
Tracer Distribution in the TM. Representative confocal images from radial (**left** two panels) and frontal (**right** two panels) plane views reveal the green tracer distribution in the high-tracer (**top** panels) and low-tracer (**bottom** panels) regions of the trabecular meshwork (TM). The blue represents DAPI staining for nuclei. DEX: dexamethasone; SC: Schlemm’s canal.

Guided by the outflow pattern, radial and frontal sections were obtained from high- or low-tracer regions. The distribution of tracers in the TM was observed in both radial and frontal sections in both groups (Figure 3).

### 2.3. DEX Treatment-Induced Changes of Extracellular Matrix (ECM) in the JCT

**Figure 4:**
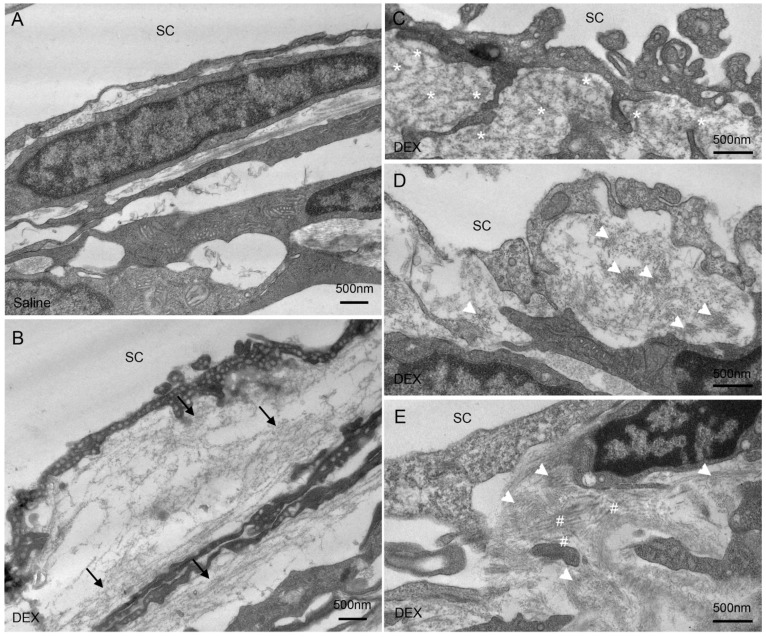
ECM Morphology in both DEX-Treated and Control Eyes. (**A**) Representative electron microscopy (EM) image shows the ultrastructure of the juxtacanalicular tissue (JCT) region in control eyes. (**B**–**D**) Representative EM images show the ultrastructure of JCT regions in dexamethasone (DEX)-treated eyes. An increased deposition of basement membrane-like materials (**B**) (black arrows), the formation of fingerprint-like arranged materials resembling basement membrane (**C**) (white asterisks), and the formation of short curly filaments (**D**) (white arrowheads) were observed. The short curly filaments were observed to occasionally mix with collagen fibers (white pound signs) and form dense materials (**E**).

**Figure 5:**
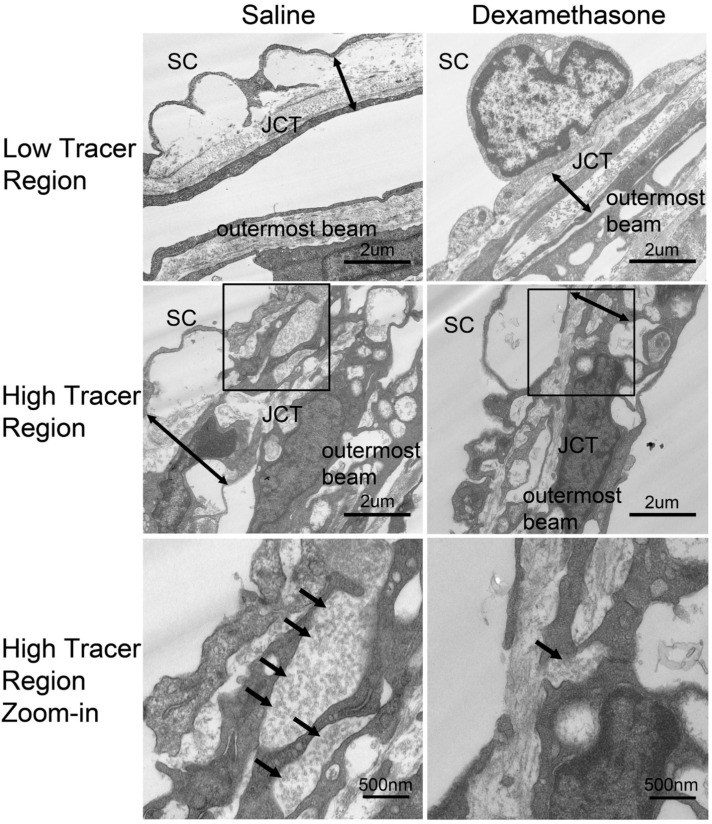
JCT in Low- and High-Tracer Regions. Representative electron microscopy images of the juxtacanalicular tissue (JCT) from low-tracer and high-tracer regions of saline control (**left** column) and dexamethasone (DEX)-treated (**right** column) eyes. The bottom row presents a larger view of the squared region in the middle row. There are more empty spaces with fluorescent tracers (black arrows) in the JCT in the high-tracer regions of control eyes. Double arrows show the JCT thickness.

Compared to control eyes, in which we found either no basement membrane (BM)-like materials deposited in the JCT (4 of 6 eyes) (Figure 4A), or only small amounts of fibrillar materials in the JCT (2 of 6 eyes), increased deposition of BM-like materials was found in all DEX-treated eyes (6 of 6 eyes) (Figure 4B). The formation of fingerprint-like arranged material (FBM) was observed in all DEX-treated eyes (6 of 6 eyes) (Figure 4C), but in none of the control eyes. The formation of short curly filaments was found in the JCT of most DEX-treated eyes (4 of 6 eyes) (Figure 4D), but in none of the control eyes. The short curly filaments were observed to mix with collagen fibers and form dense materials occasionally (Figure 4E). Both the increase in BM-like materials and formation of FBM and/or short curly filaments were observed in high- and low-tracer regions, while they were more abundant in low-tracer regions. The JCT had greater expansion, with more open spaces filled with tracer in the high-tracer regions of control eyes and became more compact after DEX-treatment (Figure 5). Specific morphometric analyses were performed to determine the changes in the thickness of JCT and percentage BM length (PBML) of the IW of SC.

### 2.4. DEX Treatment Decreased JCT Thickness

**Figure 6:**
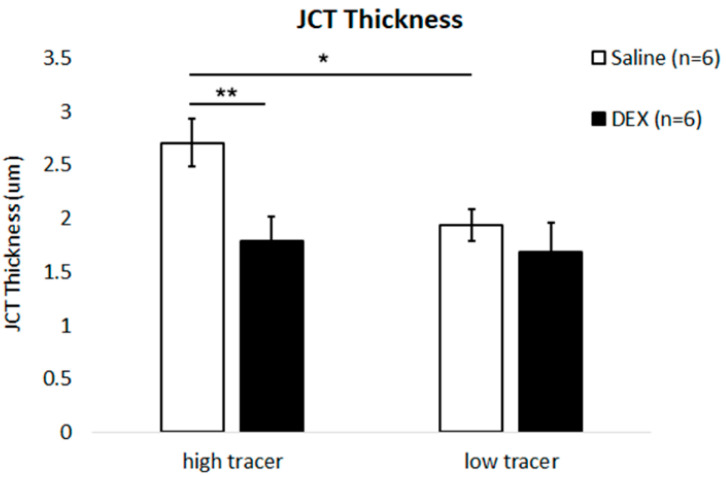
JCT Thickness. A more expanded juxtacanalicular tissue (JCT) was found in high-tracer regions compared to low-tracer regions in control eyes (**: *p* < 0.01). Dexamethasone (DEX) treatment significantly reduced JCT thickness in high-tracer regions compared to high-tracer regions in control eyes (*: *p* < 0.05).

The JCT thickness was greater in high-tracer regions (2.71 ± 0.22 µm) than in low-tracer regions (1.94 ± 0.15 µm, *p* = 0.03; Figure 5 and Figure 6) of control eyes. No significant difference in JCT thickness was found between high- and low-tracer regions in DEX-treated eyes (*p* = 0.69). In DEX-treated eyes, mean JCT thickness in low-tracer regions was similar to control eyes (1.67 ± 0.27 µm, *p* = 0.13; Mann-Whitney test), while JCT thickness in high-tracer regions of DEX-treated eyes was significantly decreased when compared to the high-tracer regions of controls (1.79 ± 0.22 vs. 2.71 ± 0.22 µm, *p* = 0.009; Figure 5 and Figure 6).

### 2.5. DEX Treatment Increased PBML of the IW of SC

**Figure 7:**
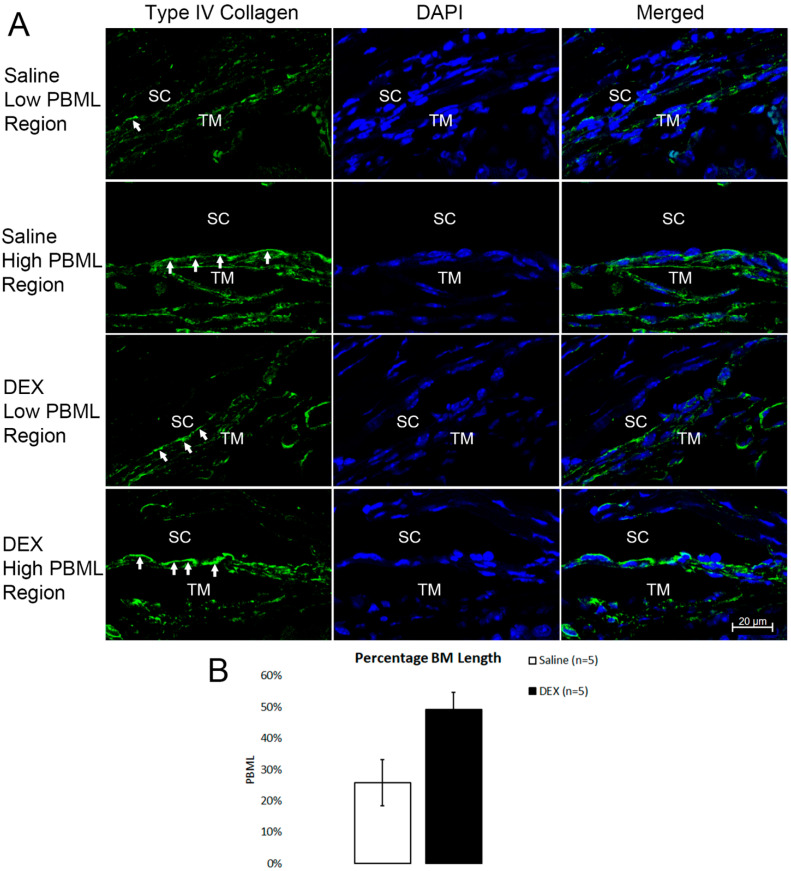
Increased PBML after DEX treatment by Immunohistochemistry. (**A**) Representative confocal images show that percentage basement membrane length (PBML) varies in both dexamethasone (DEX)-treated and untreated eyes. The green represents positive staining of type IV collagen, a major component of BM in the trabecular meshwork (TM), while the blue represents nuclei. The white arrows indicate the BM of the inner wall of Schlemm’s canal (SC). (**B**) Mean PBML was higher in DEX-treated eyes when compared to controls, indicating that BM became more continuous after DEX-treatment. However, this difference did not quite reach statistical significance (*p* = 0.06).

We first calculated the PBML of the IW of SC in the eyes without tracer injection by using immunohistochemistry staining for type IV collagen, a major component of BM. A large variance was seen in both saline and DEX groups (Figure 7A). However, there was a trend towards a greater mean PBML in DEX-treated eyes (49.19 ± 5.54%, range: 36.73–65.16%) compared to controls (25.69 ± 7.37%, range: 7.04–49.72%). This difference approached but did not reach statistical significance (*p* = 0.06; Mann-Whitney test) (Figure 7B).

**Figure 8:**
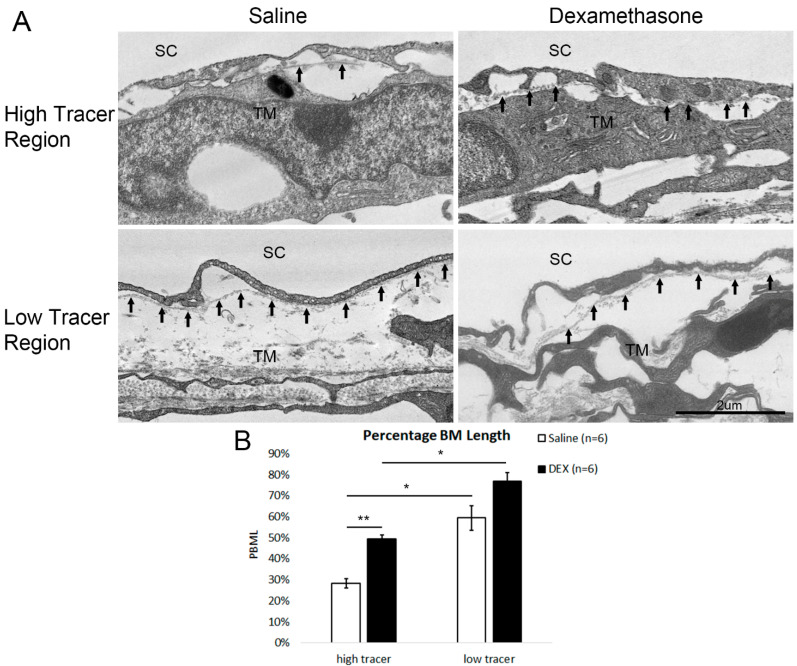
Increased PBML after DEX Treatment by Electron Microscopy. (**A**) Representative electron microscope images around the inner wall of Schlemm’s canal (SC) from high- and low-tracer regions of saline- and dexamethasone (DEX)-treated eyes. Black arrows indicate the basement membrane (BM), or fibrillary BM-like structure formed after DEX treatment. (**B**) In both saline- and DEX- treated eyes, mean percentage BM length (PBML) is significantly greater in low-tracer regions compared to high-tracer regions (*: *p* < 0.05; **: *p* < 0.01). DEX treatment induced a significant increase in mean PBML in high-tracer regions compared to that in high-tracer regions in control eyes (*: *p* < 0.05).

Since the eyes used for type IV collagen staining were not labeled with tracer to distinguish high- and low-flow regions, PBML was further analyzed in high- and low-tracer regions by electron microscopy in the tracer-injected eyes. In saline-treated eyes, BM often appeared discontinuous in high-tracer regions, while more continuous BM was observed in low-tracer regions (Figure 8A). In DEX-treated eyes, a more continuous BM was observed in both high- and low-tracer regions (Figure 8A). Moreover, as previously mentioned, increased fibrillary BM-like structures were found in DEX-treated eyes. These fibrillary BM-like structures can be difficult to distinguish from the regular BM when located near the basal lamella of IW cells. Therefore, some BM-like structures may be included as BM in measurements. In saline-treated eyes, PBML was significantly greater in low-tracer regions compared to high-tracer regions (59.34 ± 5.98% vs. 28.27 ± 2.12%, *p* = 0.03; Wilcoxon signed-rank test) (Figure 8B). In DEX-treated eyes, the difference in PBML between low-tracer (76.99 ± 3.81%) and high-tracer regions (49.47 ± 1.88%) was also significant (*p* = 0.03). In DEX-treated eyes, PBML in low-tracer regions was not significantly different from control eyes (76.99 ± 3.81% vs. 59.34 ± 5.98%, *p* = 0.06; Mann-Whitney test), while PBML in high-tracer regions was significantly greater compared to controls (49.47 ± 1.88% vs. 28.27 ± 2.12%, *p* = 0.002; Figure 8B).

### 2.6. Overall JCT Thickness and Overall PBML

**Figure 9:**
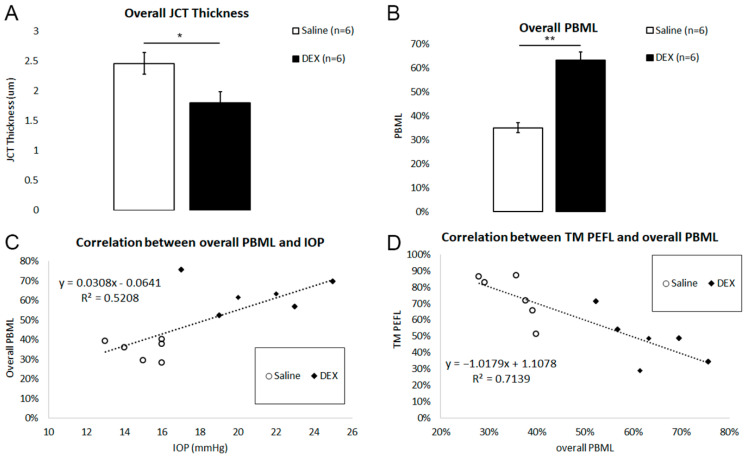
Overall JCT Thickness and Overall PBML. (**A**) The overall juxtacanalicular tissue (JCT) thickness was significantly decreased in dexamethasone (DEX)-treated eyes when compared to controls (*: *p* < 0.05). (**B**) Mean overall percentage basement membrane length (PBML) was significantly increased in DEX-treated eyes when compared to controls (**: *p* < 0.01). (**C**) There was a significant positive correlation between IOP and overall PBML (*R*^2^ = 0.52, *p* = 0.008). (**D**) There was a significant negative correlation between TM PEFL and overall PBML (*R*^2^ = 0.71, *p* = 0.0005).

We used TM PEFL to estimate the overall JCT thickness (JCT thickness_overall_ = JCT thickness_high-tracer region_ × TM PEFL + JCT thickness_low-tracer region_ × (1 − TM PEFL)) and the overall PBML (PBML_overall_ = PBML_high-tracer region_ × TM PEFL + PBML_low-tracer region_ × (1 − TM PEFL)). The overall JCT thickness was significantly decreased after DEX treatment when compared to controls (1.80 ± 0.18 µm vs. 2.46 ± 0.18 µm, *p* = 0.04; Figure 9A). Overall PBML was significantly increased after DEX treatment when compared to controls (63.18 ± 3.45% vs. 34.94 ± 2.10%, *p* = 0.002; Figure 9B). Overall PBML is positively correlated with IOP (*R*^2^ = 0.58, *p* = 0.008; Figure 9C) but negatively correlated with TM PEFL (*R*^2^ = 0.71, *p* = 0.0005; Figure 9D).

### 2.7. Effects of DEX Treatment on Body Weight (BW)

At the endpoint of treatment (Week 5), BW was 22.7 ± 0.7 g in DEX-treated male mice, which was a 3% average decrease (range: −13% to +8%) from their baseline (23.4 ± 0.5 g) and an average 4% less than male controls (23.6 ± 0.5 g). BW was 17.1 ± 0.3 g in DEX-treated female mice, which was a 4% average decrease (range: −11% to 0%) from their baseline (17.9 ± 0.2 g) and an average 13% less than female controls (19.6 ± 0.3 g).

## 3. Discussion

In this study, we used an in vivo mouse model to investigate the effects of five weeks of topical steroid (DEX) treatment on IOP, outflow pattern, and morphology. To the best of our knowledge, this is the first in vivo study to investigate the DEX-induced outflow pattern change and its morphological correlations. All of the DEX-treated mice had at least one eye showing a ≥3 mm Hg increase in IOP during DEX treatment. Therefore, 100% of the mice in this study are considered as steroid responders. Our results are consistent with the previous two studies that showed either no evidence for the presence of steroid “nonresponses” in mice by Overby et al. [24] or 90–95% of mice developed SIOH after DEX treatment (Zode et al.) [27].

We found that DEX treatment induced ~4 mm Hg increase in IOP. As we hypothesized, this steroid-induced IOP elevation was associated with reduced active filtration areas in the TM (~1/3 reduction in TM PEFL). There was a significant negative correlation between TM PEFL and IOP. Further investigation of ultrastructural changes in the JCT region showed that steroid treatment caused a decrease in the JCT thickness in high-tracer regions. Additionally, we found abnormal accumulation of ECM in the JCT of DEX-treated eyes, including BM-like materials, FBM, short curly filaments, and more continuous BM of the IW of SC. These data support our hypothesis that the elevated IOP induced by steroid administration is associated with a reduction in active filtration area caused by morphological changes in the JCT region of the TM in mouse eyes.

### 3.1. Correlations between IOP, TM PEFL, and PBML

Similar to our previous study on ocular hypotensive SPARC-null mice, we found an inverse relationship between IOP and TM PEFL. Although this relationship was statistically significant (*p* = 0.002), the correlation between IOP and TM PEFL (*R*^2^ = 0.38; Figure 2C) was much weaker when compared to the correlation between IOP and TM PEFL reported in the previous study (*R*^2^ = 0.72) [52]. Meanwhile, although we found a strong negative correlation between TM PEFL and overall PBML (*R*^2^ = 0.71; Figure 9D), the positive correlation between IOP and overall PBML was also relatively weaker (*R*^2^ = 0.52; Figure 9C). There are two possible reasons for these weak correlations between IOP and either TM PEFL or PBML in the current study. First, while the endpoint IOP was measured under less than 3 min anesthesia by isoflurane, the fluorescent tracers were injected into the mouse eyes and penetrated the TM when the mice were deeply anesthetized by ketamine + xylazine, with the whole procedure taking ~1 h. General anesthetics are known to reduce IOP, which falls further as deeper levels of anesthesia are attained. The decrease in IOP is attributed to an increase in outflow facility [54], relaxation of the extraocular muscles [54], and a decrease in the rate of aqueous humor formation [55,56]. However, this IOP reduction induced by general anesthetics seems more significant on rodents with elevated IOP when compared to normal IOP controls [27,57,58]. Therefore, the actual IOP of steroid-treated eyes during tracer injection may decrease more when compared to the control eyes, causing a weaker correlation between the IOP measured before deep anesthesia and TM PEFL/BM continuity. Secondly, the current study focused on the effects of DEX on the trabecular outflow, but the influence of uveoscleral outflow may not be negligible. The TM PEFL and BM continuity are supposed to correlate with trabecular outflow facility (C_trabecular_). The assumption of linear correlation between IOP and trabecular outflow facility was based on the modified Goldmann equation (Flowrate_total_ = (IOP − P_ESV_) C_trabecular_ + Flowrate_uveoscleral_). This equation has an assumption that uveoscleral flow rate is independent from IOP. However, direct tracer-based measurements indicate that uveoscleral outflow is not entirely pressure-independent, although it is relatively pressure-insensitive [59,60,61,62]. In addition, uveoscleral outflow may account for a much greater portion of the total aqueous outflow in mouse eyes [63,64]. Therefore, IOP-dependent influence on uveoscleral outflow may have also contributed to a weaker linear correlation between IOP and TM PEFL/PBML, especially when eyes with elevated IOP are involved.

The average TM PEFL in control eyes calculated in the current study was greater than that calculated in the SPARC-null mice study (75.09 ± 3.60% vs. 54.68 ± 9.95%) [52]. This may be due to the more advanced confocal microscope (Zeiss LSM 700 vs. Zeiss 510 Axiovert M100), operation software (ZEN2010 vs. Zeiss LSM 510), and imaging software (ZEN blue 2.3 vs. Zeiss LSM 510) used in this study when compared to the previous study, which may have allowed the visualization of more fluorescent tracers in the TM. Moreover, in order to clearly show the edge of radial wedges in the background, 120 of white balance was used for the analyses, which was set at a greater value in the previous study [52] to ensure a black background. Our TM PEFL results in control eyes were similar to the TM PEFL in ex vivo perfused human donor control eyes (75.09 ± 3.60% vs. 72.19 ± 5.72%) [65].

### 3.2. Systemic Effects of Steroids Were Observed in Mice

Although this study aimed to investigate the effects of topical steroid on mouse eyes, we would expect that some systemic effects of steroids were also involved due to the self-grooming behavior of rodents and possible diffusion of steroid into the circulatory system. A systemic administration of steroid causes BW loss in mice [24,26]. About 40% of the DEX-treated mice were eliminated from one of the previous systemic steroid studies because more than 20% BW loss was found in these mice by 3–4 weeks [24]. In the current study, the steroid-treated group exhibited 3–4% (with a maximum of 13%) BW loss when compared to baseline, indicating much weaker systemic effects of steroid treatment. None of the animals in the current study were found in critical health condition due to BW loss and needed to be removed from the study. Furthermore, systemic administration of steroids is the least likely route to cause IOP elevation in the clinic [10]. Therefore, the topical steroid model is a more effective model for mechanistic and therapeutic studies of SIOH. Recently, Patel et al. reported a novel method to introduce steroid into a mouse’s eye by using periocular conjunctival fornix injections, which may cause the elevation of IOP without decreasing BW [25]. If we combine the BW data from male and female mice, at Week 5, BW of the DEX-treated mice from both sexes was 19.7 ± 0.8 g, with no significant difference from BW of the saline-treated mice from both sexes (21.5 ± 0.6 g, *p* = 0.09). This is consistent with the results reported by Patel et al., with no indication of the sex of mice used for BW measurements [25]. However, we found this difference is significant in female mice (17.1 ± 0.3 g vs. 19.6 ± 0.3 g, *p* = 0.008) but not in male mice (22.7 ± 0.7 g vs. 23.6 ± 0.5 g, *p* = 0.39). Our data indicate that there are still some systemic effects that are more significant in female mice, even when the steroid was topically applied.

### 3.3. Steroid-Induced Ultrastructural Changes in the TM Compared to Previous Studies

The major ultrastructural changes found in the TM associated with steroid treatment were more compacted JCT in high-tracer regions and an increased deposition of ECM. The latter is consistent with previous TM cell culture studies on the ECM effects of steroids [5,33,66,67]. FBM and BM-like materials were previously reported in steroid-treated human eyes [4,8,68], juvenile glaucoma [69], and uveitic glaucoma [70]. Unlike between the trabecular lamellae [69,70] or near corneoscleral meshwork [4], as previously reported in human eyes, we found that FBM were primarily located in the JCT, similar to studies in steroid-treated bovine eyes [36] and systemic steroid-treated mouse eyes [24]. These morphological correlations likely contribute to increased outflow resistance and result in reduced EFA. Although the composition of the BM-like materials that we observed to have increased in the DEX-treated eyes is unclear, a previous study had reported the increase of type IV collagen in the TM of steroid-induced glaucomatous eyes [71]. We hypothesize that the BM-like materials may contain similar components to BM, such as type IV collagen, perlecan, laminin, integrins, etc., due to the structural similarity to the BM in TEM images. Further immuno-labeling studies will be needed to confirm this hypothesis. Although “plaques” were previously reported in human eyes with POAG [4,72,73], juvenile glaucoma [69], uveitic glaucoma [70], and steroid-treated human [68] and bovine eyes [36], we did not observe the formation of “plaques” in the steroid-treated mouse eyes. Our finding is consistent with two previous studies: one in steroid-treated human eyes [4] and another in systemic steroid-treated mouse eyes [24]. We found the formation of a unique structure, short curly filaments, in the JCT of DEX-treated mouse eyes. Although they are much less abundant compared to BM-like materials, the composition of these filaments may need to be studied further.

A recent study found that the increased flow resistance and stiffness in glaucomatous eyes is located immediately below the surface of the IW of SC, suggesting that BM of the IW may contribute to this increased resistance [39]. Although BM of the IW is discontinuous [74,75,76], increased BM continuity has been previously reported in a systemically induced steroid ocular hypertensive mouse model [24]. Our observation of both increased accumulation of BM-like materials and increased BM continuity was consistent with a previous study on systemic DEX-induced ultrastructural changes in mouse eyes [24].

The increase in PBML may imply the increase of cell-matrix connections between the IW of SC to the ECM in the JCT, which may elevate the outflow resistance. This is consistent with a previous report showing that DEX induces focal adhesions in cell culture, via which cells connect to the matrix [77]. By labeling the outflow pattern in this current study, we found a reduction in PEFL associated with an increase in overall PBML after DEX treatment. A previous study also provided evidence of the increased PBML in human steroid-induced glaucomatous eyes [24]. This previous study reported a significant negative correlation between outflow facility of ex vivo perfused systemic DEX-treated mouse eyes and PBML, whereas we found a negative correlation between TM PEFL and overall PBML, which is consistent with our previous finding that PEFL is positively correlated with outflow facility [41,44,45]. The previous systemic DEX study observed reduced empty space in the TM of DEX-treated eyes [24]. In our current study, with the association of more compacted or thinner JCT and increased deposition of ECM, we would also expect reduced empty space in DEX-treated eyes. This can also cause an increase in outflow resistance in the JCT.

However, while the previous study in systemic DEX-treated eyes indicated that immunohistochemistry staining of type IV collagen was more continuous along the IW of SC when compared to controls, we found a relatively large variance in PBML in both DEX-treated and control eyes by type IV collagen immunohistochemistry staining (Figure 7). Based on our PBML measured with TEM images, the type IV collagen staining along the IW varies depending on the flow type (high- or low-tracer) of the area. However, because the immunohistochemistry analysis in our current study was performed on the eyes without tracer injection, we could not directly correlate the type IV collagen staining with outflow dynamics. Further study with immunostaining of the tracer-injected eyes is required to investigate whether more continuous type IV collagen staining is correlated with low-tracer regions.

### 3.4. Mechanisms of Steroid-Induced IOP Elevation

Although further investigation to understand more detailed mechanisms may be needed, our data demonstrated that DEX may increase outflow resistance in the JCT via increasing ECM deposition and compacting JCT structure. Our data also suggest that steroid treatment changes morphological parameters, such as JCT thickness and PBML, more dramatically in high-tracer regions. The accumulation of these morphological changes in high-flow regions may turn some of them into low-flow regions and result in a reduction of EFA. In terms of the molecular mechanisms of steroid-induced ocular hypertension, a recent study showed that Smad3 deficiency resulted in complete inhibition of steroid-induced ocular hypertension in mouse eyes [78]. Smad3 is a protein downstream of the TGF-β2 signaling pathway. TGF-β2 is recognized as a central player in the pathology of glaucoma, stimulating ECM protein expression and secretion [79,80], which is consistent with our findings.

One limitation of this study was that the ultrastructural analysis used two radial sections (one from high- and one from low-tracer regions), which only covered a small fraction of the total TM. Additionally, the sample size (*n* = 6) was relatively small. The other limitation was that an additional group of mice with a higher dose of DEX may elucidate whether the DEX-induced hydrodynamic and morphological changes are dose-dependent.

In summary, the topical DEX treatment increased IOP in mouse eyes by reducing active filtration area in the trabecular outflow pathway. Morphological correlations with this reduction included compacted JCT in high-tracer regions and increased deposition of ECM in the JCT. Similar ultrastructural changes were found in our topical DEX-treated mouse eyes when compared to both systemic DEX-treated mouse eyes and human eyes with steroid-induced glaucoma.

## 4. Materials and Methods

### 4.1. Animal Husbandry

All experiments were completed in compliance with the Association for Research in Vision and Ophthalmology (ARVO) Statement for the Use of Animals in Ophthalmic and Vision Research. Local Institutional Animal Care and Use Committee (IACUC) approval was obtained. Thirty C57BL/6 mice (six-week-old) were purchased from Charles River Laboratories (Wilmington, MA, USA). All mice were housed in the Animal Science Center of the Boston University Medical Campus, with a 12 h light/12 h dark cycle and access to food and water *ad libitum*. Upon arrival, mice were examined to confirm a normal appearance, i.e., free of any signs of ocular disease, and allowed to acclimatize for at least three days before experiments. Since BW has been reported to decrease in mice after systemic steroid administration [24,26], BW was measured at baseline and then weekly to monitor for possible systemic effects induced by topical administration of DEX.

### 4.2. Eye Drops Administration

Thirty mice were randomly divided into two groups: a DEX-treated group and a saline-treated control group (*n* = 15 each, including 7 males and 8 females). A small eye drop (∼10 μL) of either 0.1% dexamethasone phosphate (Henry Schein Inc., Melville, NY, USA) or sterile saline (Teknova Inc., Hollister, CA, USA) (both containing 9.4 mg/mL hydroxyethyl cellulose) was topically applied to both eyes of each mouse twice daily for five weeks. The initial daily dose was given between 9 am and 11 am, with the second dose between 5 pm and 7 pm.

### 4.3. IOP Measurement

IOP was measured at baseline and then weekly between 9 am and 11 am (prior to the application of the first dose of eye drops) using a rodent rebound tonometer (Icare^®^ TONOLAB; Icare, Vantaa, Finland). Mice were initially anesthetized with 5% isoflurane in an anesthesia chamber for ~40 s and then attached to a nose cone with constant flow of 2.5% isoflurane for IOP measurements. The IOP for each eye was the median of three readings (each reading was averaged from six independent measurements by the tonometer). Mice were anesthetized for a very short time (average: 2 min 10 s, maximum: 2 min 30 s) because longer isoflurane exposure rapidly decreases IOP, especially in DEX-treated mice [27,57].

### 4.4. Tracer Injection

At the end of treatment, mice were anesthetized with 87.5 mg/kg ketamine (Henry Schein Inc., Melville, NY, USA) and 12.5 mg/kg xylazine (Henry Schein Inc.) by intraperitoneal injection. One eye of each mouse was then injected with fluorescent tracers as previously described [52]. In brief, a 10 μL Hamilton microsyringe (Nanofil; World Precision Instruments, Sarasota, FL, USA) was loaded with 1 μL solution of 20 nm tracers (Ex/Em: 505/515, 2%, Invitrogen, Carlsbad, CA, USA) diluted 1:50 in Dulbecco’s phosphate-buffered saline (*v*/*v*), as well as 2 μL modified Karnovsky’s fixative (2.5% glutaraldehyde and 2% paraformaldehyde, pH 7.3) separated by a 0.2 μL air bubble. A 35G needle (NF35BL-2; World Precision Instruments) connected to this syringe was then inserted into the anterior chamber centrally to optimize uniform distribution. The tracer volume was delivered at 4 nL/s by a microprocessor-based microsyringe pump controller (Micro4; World Precision Instruments). The 12 o’clock position of the eye was marked using TMD Tissue Marking Dye (Triangle Biomedical Sciences, Durham, NC, USA) to provide orientation. After the injection of tracers, 45 min were allowed for the tracers to migrate through the anterior chamber, penetrate the TM, and reach SC, while the needle remained in the eye. During this time, artificial tears (Henry Schein Inc.) were applied to the cornea of both eyes to prevent dehydration. To prevent blood reflux into SC when the needle was withdrawn, modified Karnovsky’s fixative (2 μL) was subsequently injected into the anterior chamber while additional fixative was simultaneously applied to the exterior of the eye for 30 min. Both eyes were enucleated after euthanasia of the mouse. The eye injected with tracers was placed in Karnovsky’s fixative while the other eye was placed in 4% paraformaldehyde at 4 °C overnight, then transferred in PBS and kept at 4 °C for further processing.

### 4.5. Confocal Microscopy

**Figure 10:**
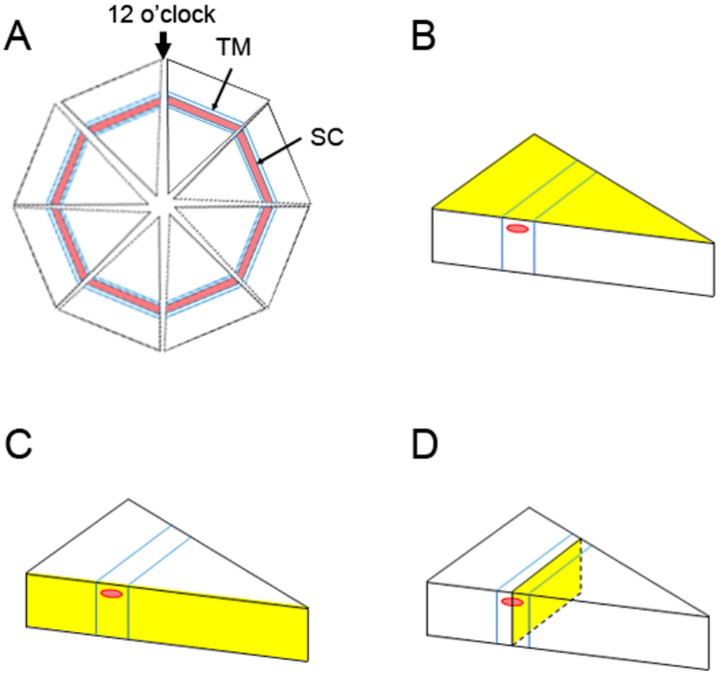
Dissection Methods and Imaging Planes. (**A**) Pattern of an enucleated eye dissected into eight radial wedges. Trabecular meshwork (TM) region and Schlemm’s canal (SC) are demarcated. Analysis of one of these eight wedges is illustrated in the following panels. The yellow surface represents the side of the tissue seen in the en face view (**B**), radial section (**C**), and frontal section (**D**). The frontal section plane usually bisects SC and the TM region, as shown here.

All tracer-injected eyes were examined using an Olympus MVX10 (Olympus, Tokyo, Japan) fluorescent stereomicroscope. Eyes with no tracer present in the TM region were excluded from further processing and analyses. Each of the successfully injected eyes (DEX-treated: *n* = 12; saline-treated: *n* = 10) were dissected into eight radial “wedges”, as shown in Figure 10A. For trabecular outflow imaging, each wedge was immersed in mounting media (Life Technologies, Carlsbad, CA, USA) in a glass-bottom dish and imaged in the en face view from the corneal side (highlighted plane in Figure 10B) with a Zeiss LSM 700 confocal microscope (Carl Zeiss, Peabody, MA, USA). The images were taken with a 10× objective and maximum pinhole (confocal slice thickness = 143 μm) to capture the entire fluorescence throughout the thickness of the TM tissue. Images were captured using the ZEN2010 operating software (Carl Zeiss).

The tissue was subsequently sectioned in both radial (Figure 10C) and frontal (Figure 10D) planes in both high- and low-tracer regions. Two radial and four frontal sections were obtained from high- and low-tracer regions of each eye. The obtained radial and frontal sections were immersed in Vectashield Antifade mounting medium with DAPI (Vector Laboratories, Burlingame, CA, USA) and imaged with a Zeiss LSM 700 to confirm the regions with high tracer or low tracer. Sections that failed to confirm with high- or low-tracer regions were excluded from further processing.

### 4.6. PEFL Analysis

**Figure 11:**
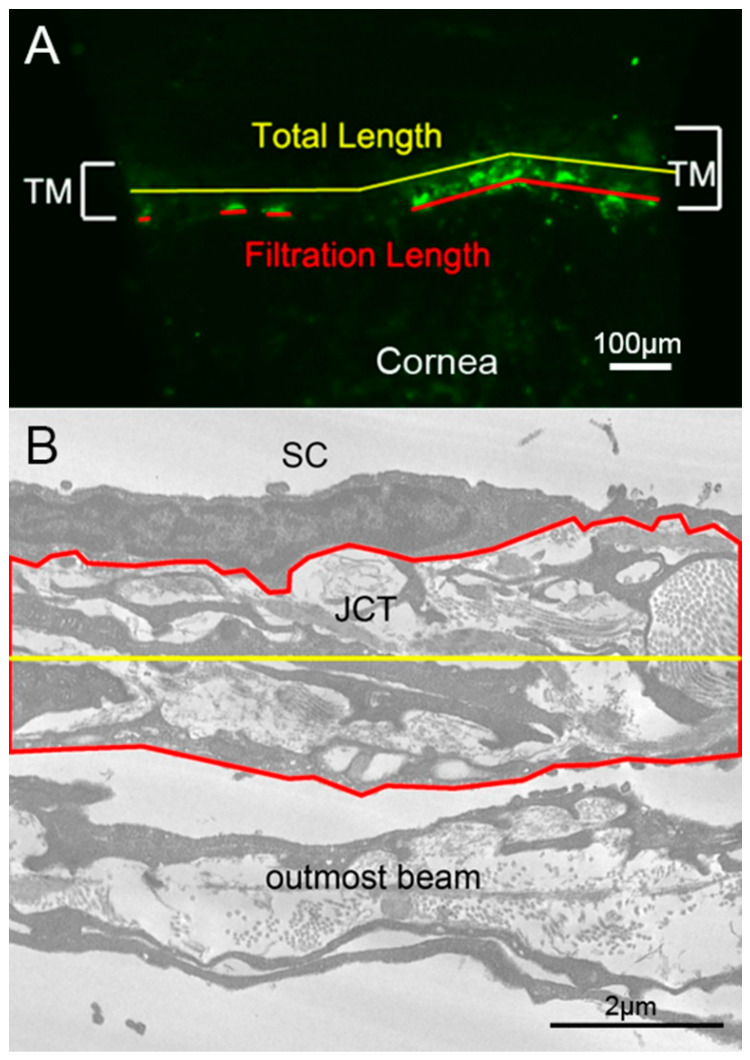
Measurement Methods for TM PEFL and JCT Thickness. (**A**) Methods of trabecular meshwork percentage effective filtration length (TM PEFL) measurements. The tracer distribution is shown in green. The red lines represent effective filtration length (FL) labeled with fluorescent tracers, while the yellow line represents the total length (TL) of trabecular meshwork (TM). The average TM PEFL in each perfused mouse eye was calculated as TM PEFL = Σ FL/Σ TL × 100%. (**B**) Methods of Juxtacanalicular Tissue (JCT) thickness measurements. JCT area (red) and JCT length (yellow) were measured in an electron microscopic image, with the average JCT thickness (Σ JCT area/Σ JCT length) calculated accordingly.

In the trabecular outflow images of each radial wedge, “total length” of the TM (TL) and “filtration length” of TM containing tracer (FL) were measured using ZEN blue 2.3 imaging software (Carl Zeiss) with white balance set at 120 and computer monitor resolution set at 1920 × 1080. The average percent effective filtration length (TM PEFL = Σ FL/Σ TL × 100%) in each eye was subsequently calculated as performed in previous studies (Figure 11A) [46,52,65]. All measurements in this study were repeated by the same investigator (RR) three months later and by another investigator (AAH) in a masked manner. The difference was less than 6% between the same investigator and less than 8% between different investigators.

### 4.7. Light and Electron Microscopy

Radial and frontal sections with high- or low-tracer presence confirmed by confocal microscopy in six randomly selected eyes from each group were processed for light and electron microscopy. Sections were post-fixed with 2% osmium tetroxide in 1.5% potassium ferrocyanide for 2 h, en bloc stained with 1.5% uranyl acetate for 90 min, dehydrated in an ascending series of ethanol and propylene oxide, and embedded in Epon-Araldite. After semithin sections (1 µm) were cut and examined with light microscopy, sections containing regions of interest were then prepared for electron microscopy (at least one radial section from high- and low-tracer regions was imaged using electron microscopy in each randomly selected eye). Ultrathin sections (80 nm) were obtained, stained with 4% methanol-based uranyl acetate to visualize extracellular matrix, and examined using a transmission electron microscope (JEOL JEM-1010, Tokyo, Japan). Images were taken along SC at original magnifications of 3000×, 5000×, and 10,000×.

### 4.8. Measurements of the JCT Thickness

JCT area was measured in 3000× electron microscopic images of radial sections by selecting the area from the basal side of the IW endothelium to the empty space adjacent to the outermost corneoscleral beams and measuring the cross-sectional area using ImageJ (NIH). JCT length was also measured by ImageJ. JCT thickness was then calculated (Figure 11B). Only the images showing a clear outermost beam were used for JCT thickness measurements. At least 30 μm length of JCT was measured for each section.

### 4.9. Immunohistochemistry

The non-tracer injected eye from each mouse was enucleated and immersion-fixed immediately with 4% paraformaldehyde for immunohistochemistry. The eyes were then paraffin-embedded and 5 μm transverse sections were obtained from five randomly selected eyes from each group. The paraffin sections were deparaffinized, microwaved in 10 mM citrate buffer (pH 6.0) for 10 min to retrieve antigen, and then stained for type IV collagen by methods adapted from Overby et al. [24]. In brief, sections were incubated for 1 h in 5% BLOTTO (Santa Cruz Biotechnology, Dallas, TX, USA) in PBS at room temperature to prevent nonspecific binding. Sections were incubated overnight at 4 °C with primary rabbit polyclonal antibody against type IV collagen (AB756P; MilliporeSigma, Burlington, MA, USA) at a dilution of 1:50 in PBS containing 2% bovine serum albumin (BSA; American Bioanalytical, Natick, MA, USA) and 0.02% Triton X-100. Sections were then rinsed three times with PBS for 10 min each and incubated for 2 h with Alexa Fluor^®^ 488 donkey anti-rabbit secondary antibody (Invitrogen, Carlsbad, CA, USA) at a dilution of 1:500 in PBS containing 0.2% BSA. Sections were rinsed in PBS and mounted with DAPI Fluoromount-G mounting medium (SouthernBiotech, Birmingham, AL, USA). Specimens were examined and imaged with Zeiss LSM 700. Negative controls were treated identically, except that the primary antibody was omitted.

### 4.10. Measurements of PBML of the IW of SC

**Figure 12:**
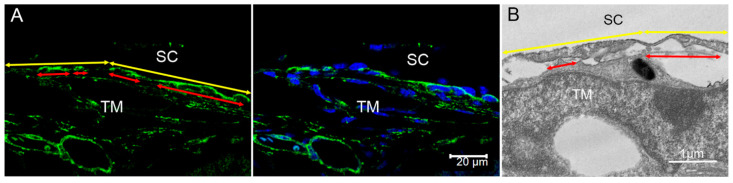
Measurement Methods of PBML of the IW of SC. (**A**) Measurement methods of Percentage Basement Membrane Length (PBML) with confocal images. The left panel shows immunohistochemical staining of type IV collagen (green) to label the BM in the trabecular meshwork (TM). The right panel shows the same image merged with DAPI staining of nuclei (blue). The yellow double arrows represent the full length of the inner wall (IW) of Schlemm’s canal (SC), while the red double arrows represent the length of BM. PBML = Σ length of BM/Σ IW length × 100%. (**B**) Measurement methods of PBML with electron microscopic images. The yellow double arrows represent the full length of the IW of SC, while the red double arrows represent the length of BM. PBML = Σ length of continuous BM/Σ IW length × 100%.

Because type IV collagen is a major constituent of BM [81,82], BM of the IW of SC can be labeled by immunohistochemistry staining of type IV collagen. Confocal images taken with a 40× oil objective were used to measure the IW length and BM length. At least 100 μm of IW length was measured in each section. PBML was calculated as Σ BM length/Σ IW length × 100% (Figure 12A). PBML was also measured using 3000× TEM images of radial sections at high- and low-tracer regions with similar methods (Figure 12B). Sections of six randomly selected eyes from each group were analyzed. At least 40 μm of IW length was analyzed in each section.

### 4.11. Statistical Analysis

A two-tailed paired and a non-paired Student’s *t*-test and three-way ANOVA were applied for IOP comparison. Mann-Whitney and Wilcoxon signed-rank tests were applied for hydrodynamic and morphological comparison between two groups. Linear regression was applied for correlation analyses. All statistical analyses used GraphPad Prism 8 (GraphPad Software, San Diego, CA, USA) with a required significance level of *p* < 0.05. All data are shown as mean ± SEM.

## Figures and Tables

**Table 1:** Comparison of Weekly IOP (mm Hg) Changes.

Time	Week 0	Week 1	Week 2	Week 3	Week 4	Week 5
Saline	14.1 ± 0.4	14.0 ± 0.3	13.5 ± 0.4	13.4 ± 0.3	13.6 ± 0.3	13.8 ± 0.3
DEX	13.8 ± 0.4	16.5 ± 0.7	16.8 ± 0.4	17.7 ± 0.5	17.8 ± 0.4	17.9 ± 0.4

## Data Availability

The raw data are readily available for presentation to the referees and the editors of the journal, if requested.
